# COVID-19 Infection in a Child Presenting With Functional Intestinal Obstruction

**DOI:** 10.7759/cureus.11448

**Published:** 2020-11-11

**Authors:** Mohammed Alsabri, Mohammed Sakr, Shatha Qarooni, Mahmoud M Hassanein

**Affiliations:** 1 Pediatrics, Brookdale University Hospital Medical Center, Brooklyn, USA; 2 Pediatric Infectious Diseases, Brookdale University Hospital Medical Center, Brooklyn, USA

**Keywords:** mis-c, sbo, covid-19

## Abstract

This report describes the case of a 13-year-old male patient presenting with functional small bowel obstruction. The child was also observed to have persistent tachycardia, and repeat cardiac examination revealed a new-onset cardiac murmur and a gallop rhythm. Acute viral myocarditis was clinically suspected, prompting further cardiac evaluations including electrocardiogram (EKG), echocardiogram (echo), and cardiac enzyme panel. Both EKG and echo findings suggested acute myocardial injury, in addition to elevated levels of cardiac enzymes and other inflammatory markers. Considering the ongoing pandemic, coronavirus disease 2019 (COVID-19) infection was suspected, but reverse transcription-polymerase chain reaction (RT-PCR) for severe acute respiratory syndrome coronavirus 2 (SARS-CoV-2) was negative. Because multisystem inflammatory syndrome in children (MIS-C) may occur later in the course of COVID-19 illness, a SARS-CoV-2 antibody test was performed, with positive results. To our knowledge, this is the first pediatric case of COVID-19 presenting as functional intestinal obstruction. We present this case to share our findings on this unique manifestation of COVID-19 with pediatric colleagues. We also engage in a brief review of MIS-C.

## Introduction

In late 2019, the novel severe acute respiratory syndrome coronavirus 2 (SARS-CoV-2), previously called 2019-nCoV, was initially reported in Wuhan, China [1]. Within weeks, global widespread infections with SARS-CoV-2 reached a pandemic scale, in response to which the World Health Organization (WHO) declared a public health emergency [1,2]. The disease that the virus causes was named coronavirus disease 2019 (COVID-19).

The first confirmed COVID-19 case in the US was recorded in January 2020 [3]. While COVID-19 infection affects all age groups and most children seem to be asymptomatic, the infection may also cause life-threatening illnesses, especially in infants aged less than one year and in children with underlying health problems [4,5,6]. COVID-19 infection in children manifests as diverse clinical findings among which fever and cough are the most commonly reported symptoms [5,6]. However, gastrointestinal (GI) symptoms may occur without respiratory symptoms [5]. Diarrhea, vomiting, and abdominal pain are the most common GI symptoms reported in children [4,5].

In April 2020, pediatricians in the UK reported cases of pediatric COVID-19 with presentations including toxic shock syndrome or incomplete Kawasaki disease [7]. Since the emergence of the pandemic, there have been increased reports of similar symptoms worldwide [5,6]. On May 14, 2020, the US Centers for Disease Control and Prevention (CDC) termed this life-threatening complication of pediatric COVID-19 as multisystem inflammatory syndrome in children (MIS-C) [8]. While it is not clear as to why some children develop MIS-C, it has been suggested to be a post-infectious inflammatory process triggering an abnormal, exaggerated immune response to the virus or antibody, or T cell recognition of self-antigens, resulting in autoantibodies [7]. MIS-C varies in severity from mild to severe and has a wide spectrum of symptoms including fever, cardiac dysfunction, abdominal pain, vomiting, diarrhea, hypotension, and conjunctival injection [5,9].

MIS-C has been described differently by the WHO, CDC, and Royal College of Pediatrics and Child Health (RCPCH). For example, WHO, unlike the CDC, does not require the child to be sick enough to be hospitalized [5,10]. In contrast, the RCPCH does not require evidence of infection or exposure to meet the case deﬁnition, similar to the CDC and WHO criteria [4]. The prevalence of GI symptoms of COVID-19 infection in children and their significance are still unknown. In fact, this is in keeping with the nature of the COVID-19 pandemic in general so far, which remains characterized by unknowns and uncertainties.

## Case presentation

In this report, we discuss the case of a previously healthy 13-year-old African American adolescent male who had been in his baseline state of health until four days before admission when he started to complain of epigastric abdominal pain, non-bilious, non-bloody vomiting, and constipation. The abdominal pain was generalized, was found to be 7/10 in intensity, constant, had no aggravating or relieving factors, and was associated with five episodes of non-bloody, non-bilious vomiting. The patient had a history of constipation and had been managed intermittently with oral laxatives. The patient denied any history of fever, skin rash, oral ulcer, decreased appetite, chest pain, cough, or difficulty in breathing. He was not taking any medications, had no known allergies, and reported no recent history of contact with sick individuals, travel, or previous surgeries.

**Figure 1:**
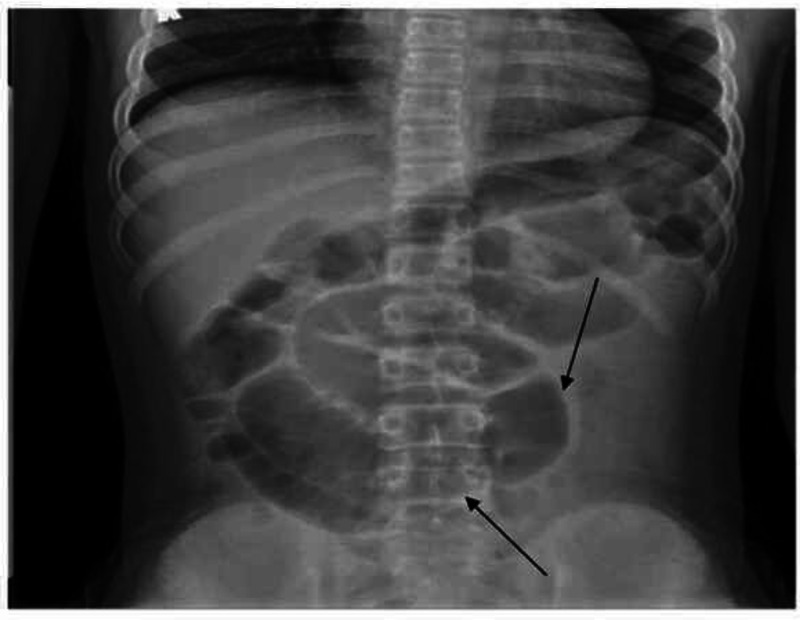
X-ray abdomen flat The image shows an abnormal bowel gas pattern with multiple abnormally dilated and air-filled loops of SB (black arrows). Partial distal SBO cannot be excluded although alternatively there may be an ileus SBO: small bowel obstruction

**Figure 2:**
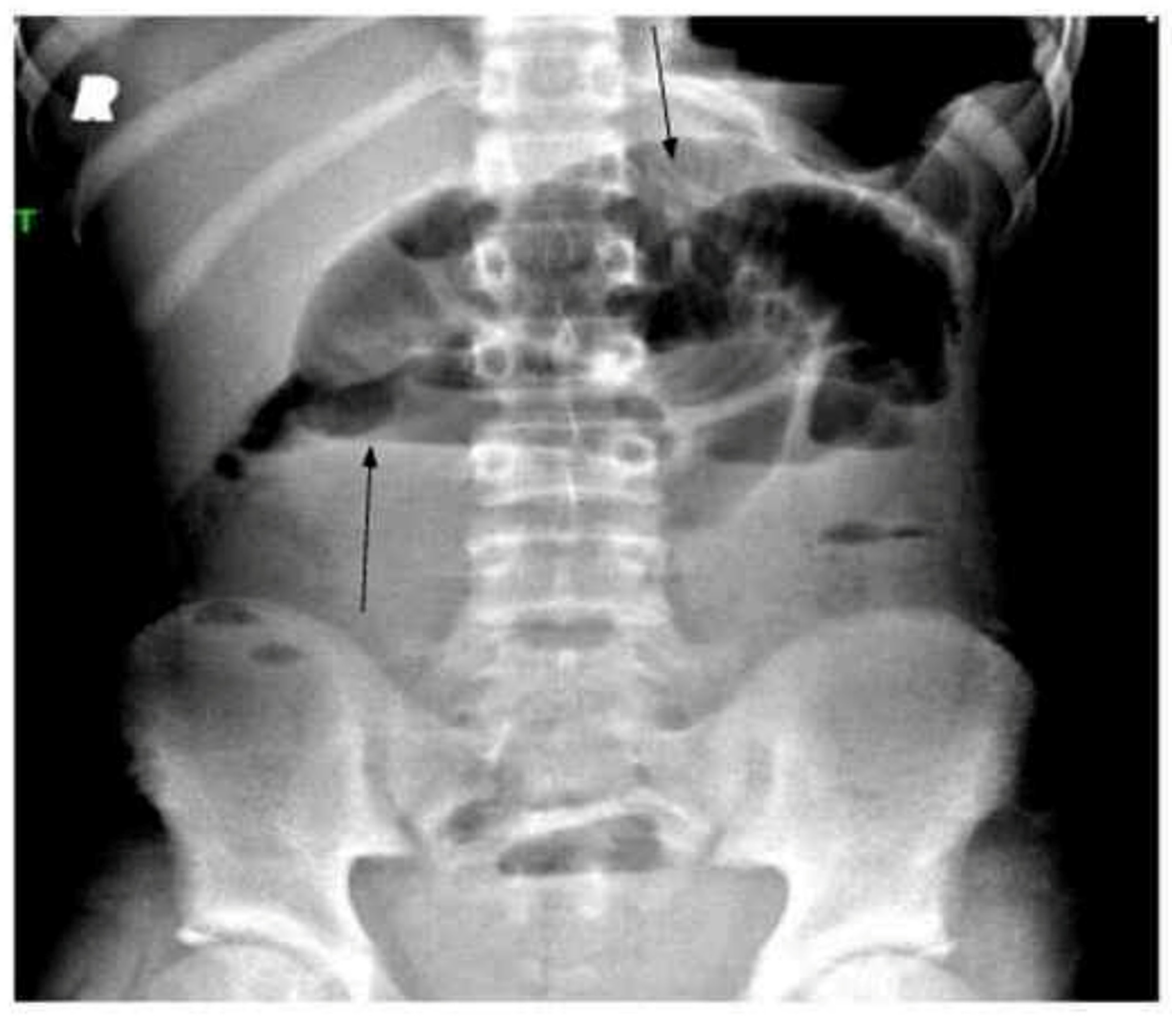
X-ray abdomen upright The image shows an abnormal bowel gas pattern with multiple abnormally dilated and air-filled loops of SB (black arrows). Partial distal SBO cannot be excluded although alternatively there may be an ileus SBO: small bowel obstruction

The patient’s vital signs were as follows: oral temperature of 36.6 °C; heart rate of 122 beats/min; respiratory rate of 18 breaths/min; blood pressure of 98/66 mmHg; and oxygen saturation of 100% on room air. On physical examination, the patient appeared well, with abdominal examination showing abdominal distention and epigastric tenderness. The findings of cardiac, pulmonary, and neurological examinations were normal. The patient had initially presented to his primary care physician, who had sent him to obtain an abdominal X-ray, which revealed a possible partial small bowel obstruction (Figures 1, 2), and had subsequently referred him to the emergency department (ED).

In the ED, the patient remained in stable condition. His vital signs were as follows: oral temperature of 36.7 °C; heart rate of 112 beats/min; respiratory rate of 22 breaths/min; blood pressure of 109/71 mmHg; and oxygen saturation of 99% on room air. The findings of the abdominal physical examination did not show any changes. Cardiac, pulmonary, and neurological examinations revealed no abnormalities. The laboratory findings showed normal levels of electrolytes (including potassium), blood gas, and lipase. The complete blood count (CBC) showed an elevated white blood cell (WBC) count to 25.1, with neutrophilic predominance and lymphopenia. The patient’s platelet count was slightly elevated (503 x 10 L). The patient’s C-reactive protein (CRP) concentration was also elevated at 27 mg/dL.

**Figure 3:**
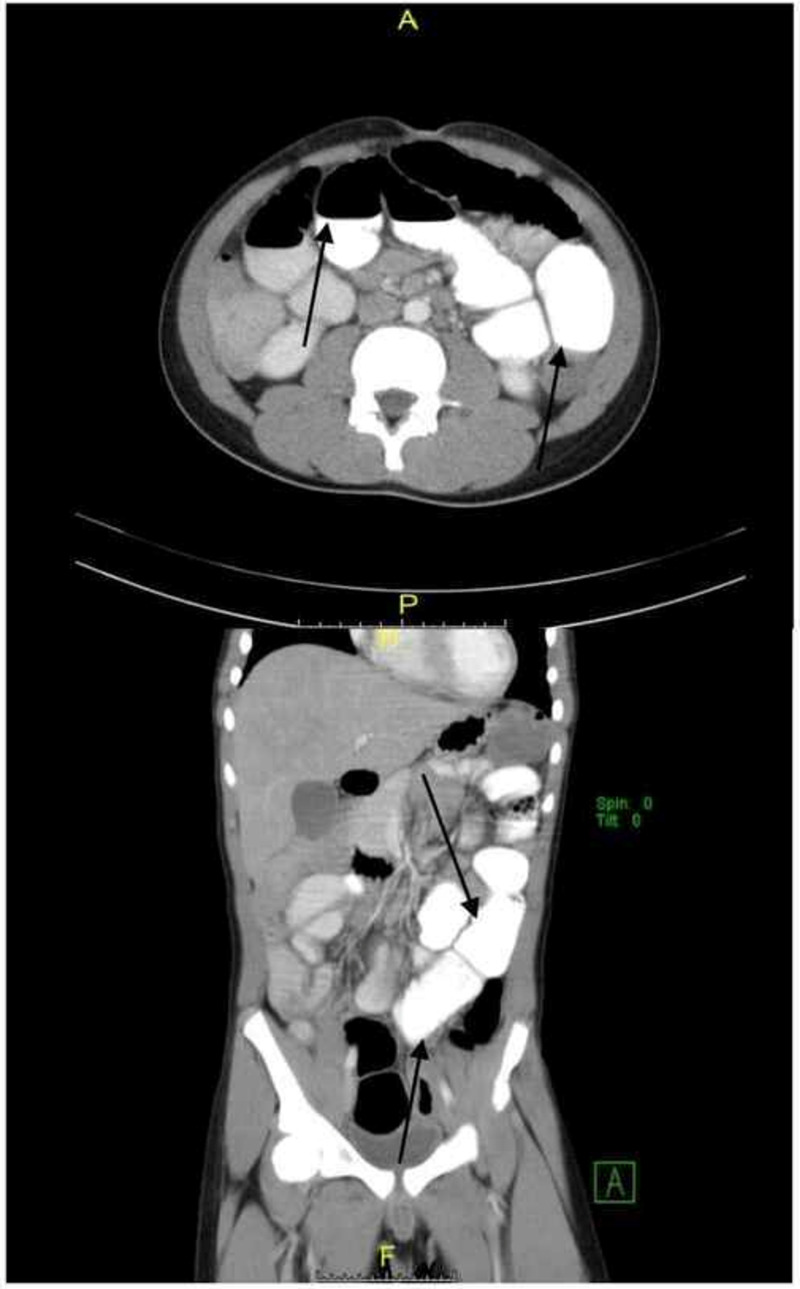
CT abdomen pelvis with contrast The image shows mild dilation of the entirety of the jejunum and ileum without a transition point and which may represent an ileus. Contrast is present throughout the small bowel, as well as in the stomach, and has reached the colon indicating there is likely no mechanical obstruction (black arrows). Continued radiographic follow-up was recommended CT: computed tomography

Abdominal CT in the ED showed mild dilation of the entirety of the jejunum and ileum without a transition point suggestive of an ileus. Contrast was present in the stomach, passing through the small bowel to the colon, ruling out mechanical obstruction (Figure 3). Given the clinical presentation and radiological findings suggestive of functional partial small bowel obstruction, the patient was admitted to the pediatric inpatient service for further evaluation of intestinal ileus. The patient was kept nil per os (NPO) and maintained on intravenous (IV) hydration. Piperacillin-tazobactam was started empirically.

As a routine procedure for all admitted patients, a nasopharyngeal specimen was collected for reverse transcription-polymerase chain reaction (RT-PCR) testing for SARS-CoV-2, the result of which was negative. A surgical evaluation ruled out acute surgical abdomen as a cause of bowel ileus with a plan for serial abdominal X-ray imaging.

On the second day of admission, the patient’s general condition remained stable. He had an oral temperature of 37.8 °C, and a respiratory rate of 18 breaths/min. However, his heart rate was persistently elevated (110-120 beats/min) and his blood pressure was below the 5th percentile for his age (91/59 mmHg), while his oxygen saturation was still 100% on room air. He had two incidents of loose non-bloody stools. On physical examination, a new holosystolic heart murmur and gallop had developed. The murmur was best heard at the cardiac apex but radiated to the whole precordium without friction rubs. Transthoracic echocardiography revealed moderate to severely depressed systolic function of the left ventricle and mildly depressed systolic function of the right ventricle, with mildly elevated right systolic pressure. Echocardiography also revealed moderate tricuspid and mitral regurgitation valve regurgitation. Electrocardiography showed sinus tachycardia with T-wave inversion at the inferior leads. Chest radiography showed cardiomegaly, bilateral pleural effusion, and cardiac lung opacities. 

In rapid progression, the patient developed a fever with a temperature of 39.4 °C, fatigue, and lightheadedness. He also complained of increasing shortness of breath, with an increased respiratory rate and decreased oxygen saturation ranging between 83% and 85% in ambient room air. He was started on a non-rebreather O_2_ mask with a flow rate of 15 L/min. To further evaluate myocardial injury associated with systemic inflammatory response, additional laboratory tests were performed. High D-dimer concentration (1,168 ng/mL), elevated erythrocyte sedimentation rate (ESR) (38 mm), and elevated cardiac enzyme levels including troponin I (initial: 0.756, repeat: 0.962 ng/mL) and proBNP (initial: 12,300; repeat: 16,200 pg/mL) were reported. Blood cultures were also collected to rule out bacterial endocarditis. 

Considering his high temperature, oxygen desaturations, progressive respiratory distress, and new-onset cardiac murmur with significantly elevated cardiac enzyme levels, the patient was transferred to another institution with a pediatric cardiac intensive care unit (PICU) in anticipation of a requirement for increased cardiac support in case of severe viral myocarditis and impending heart failure. Meanwhile, the patient was started on intravenous vancomycin and rifampin until infective endocarditis was ruled out.

Given the current epidemic of COVID-19 and the constellation of signs and symptoms, including respiratory, cardiac, and GI involvement with elevated levels of inflammatory markers and cardiac enzymes in this previously healthy child, myocarditis secondary to COVID-19 infection was suspected despite the negative nasopharyngeal test results.

At the other facility, the patient received one dose of tocilizumab and was continued on furosemide and enoxaparin. Antibiotics were discontinued due to negative blood cultures. Serial echocardiograms indicated gradual improvement of biventricular function, and repeat ECGs were within normal limits. Troponin, BNP, and D-dimer levels started to trend downward following an initial elevation. The patient was kept NPO until he was able to maintain normal oxygen saturation in ambient room air. His abdominal pain improved gradually. Oral feeding was resumed and was well-tolerated. Patient samples eventually tested positive for SARS-CoV-2 immunoglobulin G (IgG) antibodies, indicating that his illness was a late manifestation of COVID-19 presenting as an MIS-C with cardiac and GI involvement.

## Discussion

As SARS-CoV-2 continues to spread across the US and worldwide, a growing body of evidence indicates that this virus causes MIS-C [1,2,4]. Clusters of children from various countries with this inflammatory syndrome have presented with acute febrile illness and evidence of multi-organ dysfunction [4,5,6]. Their laboratory features have included neutrophilia, lymphopenia, and elevated levels of CRP and other inflammatory markers. The clinical features have included dermatologic, mucocutaneous, and GI manifestations associated with cardiac dysfunction [4,5]. The present case had similar laboratory parameters and some consistent clinical features. However, the patient's clinical presentation was dominated by abdominal pain, constipation, distension, and clinical features suggestive of small bowel obstruction with radiographically proven ileus (Figures 1, 2). To our knowledge, this is the first report of a child infected with SARS-CoV-2 with evidence of MIS-C presenting with functional intestinal obstruction due to paralytic ileus.

Coronaviruses are large, enveloped, positive-strand RNA viruses that can be divided into four categories: genera-alpha, beta, gamma, and delta; of these four, alpha and beta coronaviruses are human coronaviruses (HCoVs) known to infect humans [1,7,9,11]. Four HCoVs (HCoV 229E, NL63, OC43, and HKU1) are endemic globally and account for 10-30% of upper respiratory tract infections in adults [1,7,11]. 

Although HCoVs have long been considered inconsequential pathogens because of their mild phenotypes in humans, the occurrence of two large-scale epidemics with alarming morbidity and mortality [severe acute respiratory syndrome coronavirus (SARS-CoV) and Middle East Respiratory Syndrome (MERS) coronavirus] in the early 21st century has changed that view [12,13]. As mentioned above, in January 2020, the WHO named the novel coronavirus as SARS-CoV-2, and the disease it causes has been termed COVID-19. This virus has spread globally on a pandemic scale. To date, this virus has infected more than 33 million people worldwide, resulting in more than one million deaths.

Our patient presented with predominantly GI symptoms with abdominal pain and distension secondary to ileus confirmed by both X-ray and abdominal CT, in addition to coagulopathy, myocardial dysfunction with cardiovascular instability, and respiratory distress requiring oxygen supplementation; this was in contrast with most cases of acute COVID-19 among hospitalized children and adults, in whom respiratory symptoms were most prominent [5]. Interestingly, our patient lacked dermatological and mucocutaneous findings. The patient progressed to warm vasoplegic shock, which responded to volume resuscitation. This constellation suggested inflammatory vasculopathy, with some similarities to Kawasaki disease, mainly the atypical type, and toxic shock syndrome.

Our patient’s clinical findings were consistent with those of other studies to date [4,5]. Recent statewide surveillance by the New York State Department of Health (NYSDOH) established to describe hospitalized patients with MIS-C has revealed that various presenting symptoms and manifestations were correlated with age [9,14]. In this study, the prevalence of mucocutaneous symptoms was highest among children aged zero to five years. Children aged 0-12 years with MIS-C were more likely to present with Kawasaki disease-like symptoms such as conjunctival injection, rash, and oral mucosal changes compared to adolescents with MIS-C. The prevalence of myocarditis was higher among adolescents [9,14]. Although the prevalence of GI symptoms was high in all age groups, none of these children presented with functional intestinal obstruction. Additionally, this surveillance also revealed that 40% of patients were black and 36% were Hispanic [14]. This may be a reflection of the well-documented elevated incidence of SARS-CoV-2 infection among black and Hispanic communities. SARS-CoV-2 PCR testing was positive in 51% of cases, while serologic assay for IgG antibodies was positive in 99% of cases. In the present case, the RT-PCR assay was negative on two occasions, but IgG antibody testing was positive, suggesting that even recent asymptomatic infections may be complicated, followed by MIS-C [4,5,7].

To date, the association between COVID-19 infection and MIS-C has not been conclusively proven. In line with studies from Italy, France, and the UK, MIS-C cases in New York State followed the peak of the COVID-19 epidemic in the state by four to six weeks, which suggests that MIS-C is probably a post-infectious immune dysregulation process related to COVID-19 and that a temporal and geographic association between COVID-19 and MIS-C is very likely [5,7]. In addition, most patients (including ours) had serological evidence of recent SARS-CoV-2 infection, which supports a laboratory-based association in addition to temporal and geographic associations. There are several explanations for the possible association between COVID-19 infection and MIS-C. In PCR-negative, antibody-positive cases, it possible that a coronavirus infection in the recent past could trigger an immune response simulating Kawasaki disease or toxic shock syndrome. COVID-19 is also known to cause excessive inflammation and cytokine storm like Kawasaki disease or toxic shock syndrome during the active infective phase; however, the exact pathophysiology of MIS-C is not yet well understood [7,15].

Our report has some limitations. The patient had not had any illness in the few months prior to his presentation or a history of contact with a COVID-19-confirmed case to suggest acute COVID-19 infection before the presentation. In addition, two consecutive nasopharyngeal swabs were negative by RT-PCR; however, most children have mild or no illness from SARS-CoV-2 infection and MIS-C has been reported to follow asymptomatic SARS-CoV-2 infection. Additionally, Kawasaki disease and toxic shock syndrome are relatively vague conditions without definitive diagnostic tests, which adds to the challenge. Similarities in laboratory values such as CRP, D-dimer, and ferritin concentrations may be clues to both diagnosis and pathogenesis; unfortunately, these laboratory tests are nonspecific. Furthermore, the sensitivity and specificity of the case definition for confirmed MIS-C may be further affected by the varying performance of both PCR and serologic tests depending on the manufacturer and setting.

## Conclusions

In conclusion, the recognition of the syndrome and early identification of children with MIS-C, including early monitoring of blood pressure, cardiac biomarkers, and electrocardiographic and echocardiographic evaluation, could inform appropriate supportive care and other potential therapeutic options, including early referral to centers with cardiac ICUs. As PCR testing possesses much higher specificity than antibody testing, we recommend more PCR testing for children presenting with mild symptoms or those who are asymptomatic but have had contact with confirmed Covid-19 cases to establish a stronger association between MIS-C and SARS-CoV-2. It is also crucial to establish surveillance for MIS-C cases, particularly in communities with higher levels of SARS-CoV-2 transmission. Additionally, an awareness of the potential negative consequences of widespread dissemination of this possible diagnosis is of paramount importance. Misdiagnosis of MIS-C could drive overtreatment, and anchoring on this diagnosis could hinder the consideration of other hyper-inflammatory or infectious conditions. False inflation of the reported incidence could further heighten anxiety and perhaps lead to public health interventions of uncertain benefits such as continued remote learning instead of school closures.

## References

[1] Lu H, Stratton CW, Tang YW (2020). Outbreak of pneumonia of unknown etiology in Wuhan, China: the mystery and the miracle. J Med Virol.

[2] Petersen E, Koopmans M, Go U (2020). Comparing SARS-CoV-2 with SARS-CoV and influenza pandemics. Lancet Infect Dis.

[3] Holshue ML, DeBolt C, Lindquist S (2020). First case of 2019 novel coronavirus in the United States. N Engl J Med.

[4] Sperotto F, Friedman KG, Son MBF, VanderPluym CJ, Newburger JW, Dionne A (2020). Cardiac manifestations in SARS-CoV-2-associated multisystem inflammatory syndrome in children: a comprehensive review and proposed clinical approach (Epub ahead of print). Eur J Pediatr.

[5] Ahmed M, Advani S, Moreira A (2020). Multisystem inflammatory syndrome in children: a systematic review. EClinicalMedicine.

[6] Feldstein LR, Rose EB, Horwitz SM (2020). Multisystem inflammatory syndrome in U.S. children and adolescents. N Engl J Med.

[7] Jiang L, Tang K, Levin M (2020). COVID-19 and multisystem inflammatory syndrome in children and adolescents. Lancet Infect Dis.

[8] (2020). Centers for Disease Control and Prevention: multisystem inflammatory syndrome in children (MIS-C) associated with coronavirus disease 2019 (COVID-19). https://emergency.cdc.gov/han/2020/han00432.asp.

[9] Dufort EM, Koumans EH, Chow EJ (2020). Multisystem inflammatory syndrome in children in New York State. N Engl J Med.

[10] (2020). World Health Organization Scientific Brief: multisystem inflammatory syndrome in children and adolescents with COVID-19. https://www.who.int/publications/i/item/multisystem-inflammatory-syndrome-in-children-and-adolescents-with-covid-19.

[11] (2020). Johns Hopkins University Coronavirus Resource Center: COVID-19 global map. Cases.

[12] de Wilde AH, Snijder EJ, Kikkert M, van Hemert MJ (2018). Host factors in coronavirus replication. Curr Top Microbiol Immunol.

[13] Paules CI, Marston HD, Fauci AS (2020). Coronavirus infections-more than just the common cold. JAMA.

[14] (2020). New York State Department of Health: COVID-19 tracker. https://covid19tracker.health.ny.gov/views/NYS-COVID19-Tracker/NYSDOHCOVID-19Tracker-Map?%3Aembed=yes&%3Atoolbar=no&%3Atabs=n.

[15] Riphagen S, Gomez X, Gonzalez-Martinez C, Wilkinson N, Theocharis P (2020). Hyperinflammatory shock in children during COVID-19 pandemic. Lancet.

